# Draft genome sequence dataset of *Latilactobacillus curvatus* PN39MY isolated from fermented vegetables

**DOI:** 10.1016/j.dib.2023.109436

**Published:** 2023-07-20

**Authors:** Eric Banan-Mwine Daliri, Toma Balnionytė, Eglė Lastauskienė, Aurelijus Burokas

**Affiliations:** a Department of Biological Models, Institute of Biochemistry, Life Sciences Center, Vilnius University, Sauletekio Ave. 7, LT, Vilnius 10257, Lithuania; b Department of Microbiology and Biotechnology, Institute of Biosciences, Life Sciences Center, Vilnius University, Sauletekio Ave. 7, LT, Vilnius 10257, Lithuania

**Keywords:** Antimicrobial resistance, Virulence genes, Average nucleotide identity, Genome annotation

## Abstract

Here we report the draft genome sequence of the *Latilactobacillus curvatus* PN39MY strain. The strain was isolated from Lithuanian traditionally (homemade) fermented cucumber. The genome consisted of 83 contigs with a total size of 1,899,018 bp, an N50 of 40562 and a GC% of 42.1. After sequence trimming, 83 contigs were annotated and 1910 genes were coding sequences. The average nucleotide identity (ANI) between PN39MY and *Latilactobacillus curvatus*_ZJUNIT8 was 99.45% identifying the strain as *Latilactobacillus curvatus*. No genes related to antimicrobial resistance or virulence factors were found. The data presented here can be used in comparative genomics to identify antimicrobial resistant genes, plasmids and/or virulence factors that may be present in related *Latilactobacillus* species. The draft genome sequence data was deposited at NCBI under Bioproject with the accession number PRJNA941180.

**Specifications Table**

**Table:** 

Subject	Microbiology
Specific subject area	Microbial genomics
Type of data	The dataset consists of sequencing data
How data were acquired	Illumina NovaSeq 6000, Unicycler v 0.4.8, Prokka v 1.14.5, VFDB (Accessed on 2023.01.14), NCBI Bacterial Antimicrobial Resistance Finder Plus (software version 3.11.2, database version 2023–02–23.1), ResFinder 4.1 (database version 2022.05.24), Comprehensive Antibiotic Resistance Database (Accessed on 2023.03.15), PlasmidFinder 2.1 (database version 2023-01-18), PathogenFinder 1.1 (Accessed on 2023.03.15)
Data format	Raw
Description of data collection	The extraction protocol described in the Qiagen Genomic DNA Handbook was followed to obtain DNA from pure cultures of *Latilactobacillus curvatus* PN39MY. The DNA was sequenced using Illumina NovaSeq 6000. The raw reads were assembled and annotated. The assembled genome was screened for plasmids, antimicrobial resistance genes, bacteriocines and virulence factors.
Data source location	Institution: Department of Biological models, Institute of Biochemistry, Life Science Center, Vilnius University City/Town/Region: Vilnius Country: Lithuania
Data accessibility	The sequencing data was deposited at the National Center for Biotechnology Information (NCBI) Genebank database with accession number JARGYE000000000. The deposited draft genome sequencing data can be accessed at https://www.ncbi.nlm.nih.gov/nuccore/JARGYE000000000

## Value of the Data

• *Latilactobacillus curvatus* is a lactic acid bacterium commonly used for food fermentation and to improve human and animal health. The sequencing data described here unveils the identity and safety-related characteristics of *Latilactobacillus curvatus* PN39MY, a valuable fermented food isolate.
• The information presented for *Latilactobacillus curvatus* PN39MY is significant because the bacterium possessed none of the antimicrobial resistance genes such as macB, vanHD, vanL, baeR, baeS, mfd, lmrC, lmrD, salA, lsaA, rpsJ, tetT, tetM, efrA, efrB, tetA, vanL, TaeA, vgaD and rpsJ present in many other *L. curvatus* isolates reported in previous studies [1].
• The sequencing data and the described microbial bioinformatics workflow can be applied in comparative genomics and in the search for virulence factors, antibiotic resistance genes as well as plasmids in related lactic acid bacteria species.

## 1 Objective

*Latilactobacillus curvatus* is commonly found in fermented foods. Meanwhile, though most of them are generally regarded as safe due to their long history of use without reported risks, they may acquire antimicrobial resistance and virulence genes from the environment which can be transferred to pathogens. For this reason, we analyzed the whole genome sequence of *Latilactobacillus curvatus* PN39MY to obtain insights into possible antimicrobial resistance genes and virulent factors it might possess after it demonstrated the best ability (among 18 other lactic acid bacteria isolated from Lithuanian homemade pickles) to generate fermented beet root with antidiabetic potentials (data not shown).

## 2 Data Description

**Fig. 1:**
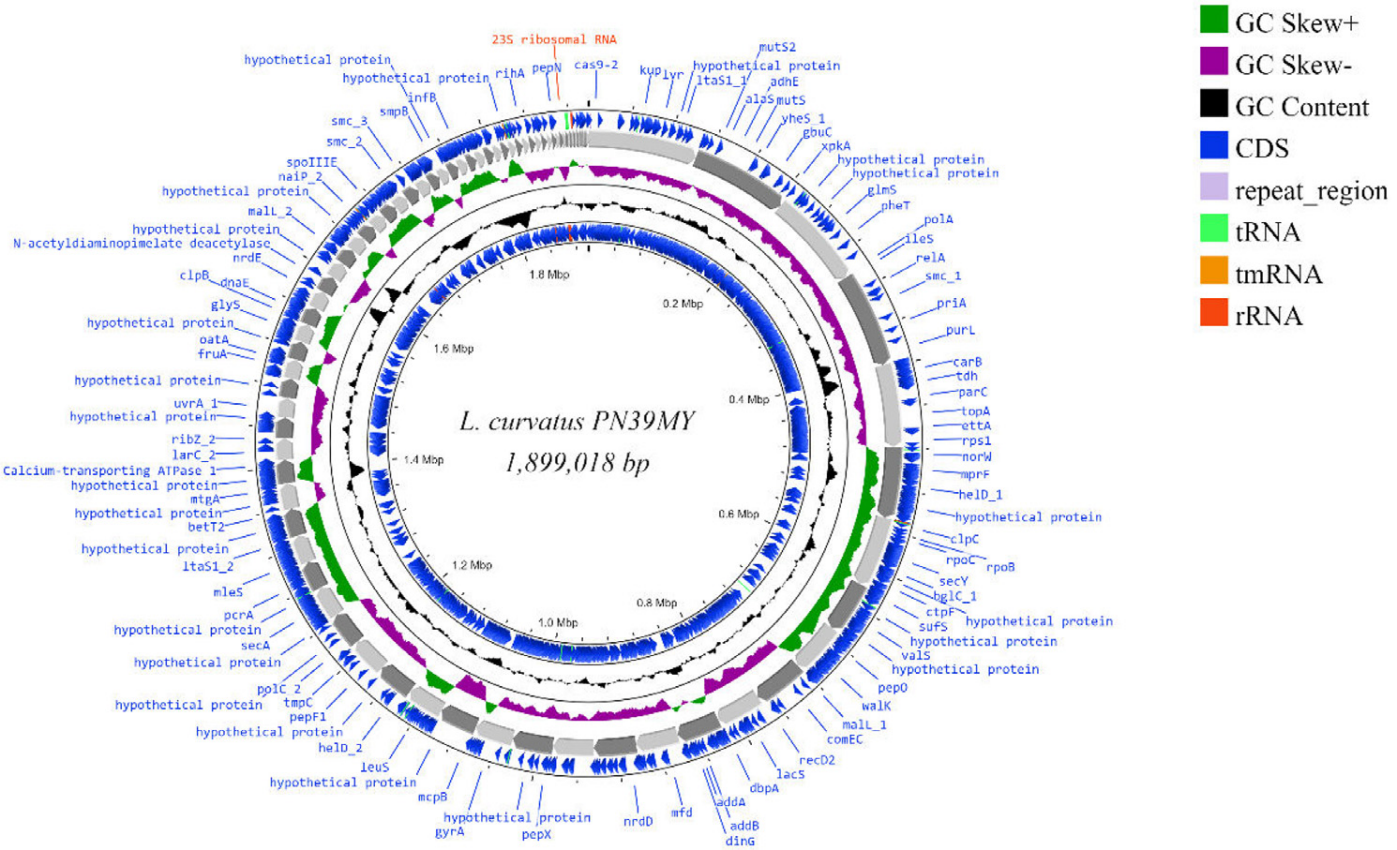
Genome map of *Latilactobacillus curvatus* PN39MY built using CGView server (https://proksee.ca/ assessed on 14th March 2023) with the predicted origin of replication at the top. The blue arrows represent CDSs; grey arrows represent the contigs, green peaks represent GC-skew+; purple represents GC-skew-; and black peaks represent G+C content.

Here, we report the draft genome sequence data of *Latilactobacillus curvatus* PN39MY including its potential antimicrobial resistance and virulence factors. The genome consisted of 83 contigs with a total size of 1,899,018 bp, an N50 of 40562 and a GC% of 42.1 (Fig. 1).

At the annotation step, a total of 83 contigs (1,899,018 bp) were assembled and annotated. The annotation generated 1975 genes of which 1910 were coding sequences (CDS), 8 ribosomal RNAs (rRNAs), 1 transfer-messenger (tmRNA), 54 transfer RNA (tRNAs) and 2 repeat regions. According to the NCBI Genome database, 74 *L. curvatus* genome assemblies have been reported [search date: 23.03.2023] with a median total length is 1,920,030 bp, a median protein count of 1844 and a median GC% of 41.9. This implies that PN39MY sequencing yielded a complete genome (98.91%) comparable in size to the median length expected. The Average Nucleotide Identity (ANI) between PN39MY and *Latilactobacillus curvatus*_ZJUNIT8 was 99.45% (supplementary data) indicating that PN39MY is a *Latilactobacillus curvatus* strain*.* The genome were screened against different antimicrobial gene databases namely; NCBI Bacterial Antimicrobial Resistance Finder Plus, ResFinder 4.1 and Comprehensive Antibiotic Resistance Database [2]. No antimicrobial resistance genes or virulent genes were identified. Also, no pathogenic factors nor plasmids were detected in the genome. The draft genome sequence dataset was deposited at the NCBI Genebank with accession number JARGYE000000000.

## 3 Experimental Design, Materials and Methods

### 3.1 DNA Extraction and Whole Genome Sequencing

The QIAGEN DNeasy PowerSoil Pro Kit was used to isolate DNA from the bacteria following the manufacturer's protocol. Briefly, the quantity of DNA in the samples were measured using the QuantiFluor® dsDNA System chemistry with the GloMax Plate Reader System. To prepare the DNA libraries, Nextera XT DNA Library Preparation Kit was used coupled with IDT Unique Dual Indexes. An amount of 1ng DNA was used as input. The genomic DNA was fragmented using Illumina Nextera XT fragmentation enzyme and a unique dual index was added to the sample. The library was then constructed using 12 cycles of PCR. The DNA library was purified using AMpure magnetic beads and eluted in QIAGEN EB buffer. The quantity of DNA in the library was measured with a Qubit 4 fluorometer and a Qubit dsDNA HS Assay Kit. The library was then sequenced with the Illumina NovaSeq platform with 2 × 150bp reads.

### 3.2 Taxonomic Identification of the Strain

**Fig. 2:**
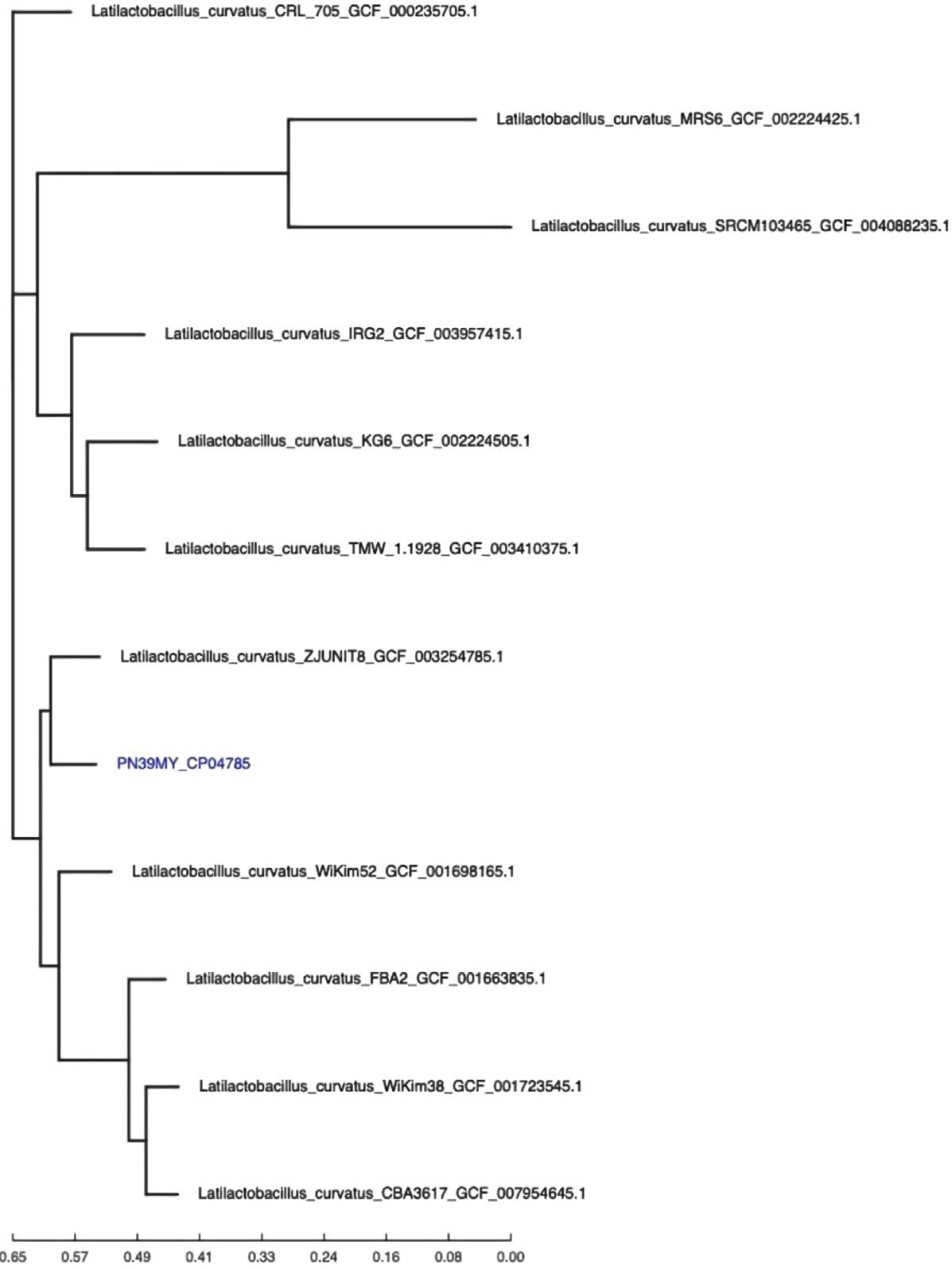
Phylogenetic tree of PN39MY bacterium. FastTree2 was used to reconstruct the phylogenetic relationship from the core-genome SNPs. The GenBank accession numbers are indicated in the phylogenetic tree.

Raw paired end reads were trimmed and processed using BBDuk, with a read quality trimming parameter of 22. SPAdes was then used to assemble the fastqs with the "–careful" parameter. The lineage_wf function in CheckM was used to assess the completeness of the assemblage. CosmosID core genome SNP typing pipeline was used to examine the assembled contigs, and to draw epidemiological conclusions based on phylogenetic placement and SNP variations. Parsnp was used as the core genome aligner to map the core genome of several microbial genomes. The final set of core-genome SNPs was used to rebuild the phylogenomic relationship among the genome using FastTree2, as shown in Fig. 2.

### 3.3 Search for Antimicrobial Resistance Genes and Virulence Factors

For antimicrobial resistance genes (AMR) and virulence factor (VF) detection, the assembled genome of PN39MY was compared to the Resfinder AMR and VFDB VF database [3] using ABRicate version 1.0.1. AMR and VF genes were considered present only if the sequences matched the assembled genome at a nucleotide identity >90% and the alignment coverage of the gene's sequence length was >60%. The average nucleotide identity (ANIm) between isolates was calculated using MUMmer [4]. Prokka Annotation Pipeline [5] was then used to annotate the genome.

### 3.4 Search for Plasmids

The contigs from the genome data were compared to the PlasmidFinder database [6,7] in order to identify any plasmids present.

## Ethics Statement

No animal or human studies were conducted in this work.

## CRediT authorship contribution statement

**Eric Banan-Mwine Daliri:** Data curation, Software, Writing – original draft, Methodology, Formal analysis, Writing – review & editing. **Toma Balnionytė:** Data curation, Software, Writing – original draft, Methodology, Formal analysis, Writing – review & editing. **Eglė Lastauskienė:** Supervision, Project administration. **Aurelijus Burokas:** Supervision, Project administration.

## Declaration of Competing Interest

The authors declare that they have no conflict of interests that could have appeared to influence the work reported in this paper.

## Data Availability

Latilactobacillus curvatus strain PN39MY, whole genome shotgun sequencing project (Original data) (GenBank).

## Appendix Supplementary materials

Image, application 1

## References

[1] Yu L., Zang X., Chen Y., Gao Y., Pei Z., Yang B., Zhang H., Narbad A., Tian F., Zhai Q., Chen W. (2022). Phenotype-genotype analysis of *Latilactobacills curvatus* from different niches: Carbohydrate metabolism, antibiotic resistance, bacteriocin, phage fragments and linkages with CRISPR-Cas systems. Food Res. Int..

[2] Alcock B.P., Huynh W., Chalil R., Smith K.W., Raphenya A.R., Wlodarski M.A., Edalatmand A., Petkau A., Syed S.A., Tsang A.R. (2023). CARD 2023: expanded curation, support for machine learning, and resistome prediction at the Comprehensive Antibiotic Resistance Database. Nucleic Acids Res.

[3] Chen L., Yang J., Yu J., Yao Z., Sun L., Shen Y., Jin Q.J.N.a.r. (2005). VFDB: a reference database for bacterial virulence factors. Nucleic Acids Res.

[4] Kurtz S., Phillippy A., Delcher A.L., Smoot M., Shumway M., Antonescu C., Salzberg S.L. (2004). Versatile and open software for comparing large genomes. Genome Biol.

[5] Seemann T.J.B. (2014). Prokka: rapid prokaryotic genome annotation. Bioinformatics.

[6] Carattoli A., Hasman H.J. (2023). PlasmidFinder and in silico pMLST: identification and typing of plasmid replicons in whole-genome sequencing (WGS). Methods Mol. Biol. 2075.

[7] Camacho C., Coulouris G., Avagyan V., Ma N., Papadopoulos J., Bealer K., Madden T.L. (2009). BLAST+: architecture and applications. BMC bioinformatics.

