# Corrigendum to: Draft genome sequence dataset of *Latilactobacillus curvatus* PN39MY isolated from fermented vegetables Data in Brief, 49 (2023) 109436

**DOI:** 10.1016/j.dib.2023.109496

**Published:** 2023-08-12

**Authors:** Eric Banan-Mwine Daliri, Toma Balnionytė, Eglė Lastauskienė, Aurelijus Burokas

**Affiliations:** a Department of Biological Models, Institute of Biochemistry, Life Sciences Center, Vilnius University, Sauletekio Ave. 7, Vilnius LT-10257, Lithuania; b Department of Microbiology and Biotechnology, Institute of Biosciences, Life Sciences Center, Vilnius University, Sauletekio Ave. 7, Vilnius LT-10257, Lithuania

The authors regret for the error in the grant information indicated in the manuscript. The correct information should be “This project received funding from the 10.13039/501100008530European Regional Development Fund (project No. 09.3.3-LMT-K-712-23-0117) under a grant agreement with the 10.13039/501100004504Research Council of Lithuania (LMTLT).

The authors would like to apologise for any inconvenience caused.

